# Resistance exercise with combined Valsalva manoeuvre acutely increases carotid-femoral pulse wave velocity in healthy untrained individuals

**DOI:** 10.1007/s00421-026-06167-z

**Published:** 2026-05-29

**Authors:** Blake G. Perry, Melissa J. Perry, Riley Dixon, Michael Bush, Helen Ryan-Stewart

**Affiliations:** 1 School of Health Sciences, Massey University, Wallace Street, Wellington, 6021 New Zealand; 2 Medical Research Institute of New Zealand, Wellington, New Zealand; 3 Faculty of Education, Humanities and Health Sciences, Eastern Institute of Technology, Napier, New Zealand

**Keywords:** Arterial stiffness, Middle cerebral artery blood velocity, Cerebrovascular response to resistance exercise

## Abstract

**Purpose:**

The Valsalva manoeuvre (VM) may contribute to the observed increase in pulse wave velocity (PWV) following resistance exercise (RE). However, VM use during RE is rarely reported or discussed in the literature. We compared the acute carotid-femoral PWV response before and following RE with paced breathing, RE with a VM (REVM) and the VM alone.

**Methods:**

15 healthy RE-untrained participants (female = 8) (mean ± SD: age, 24 ± 5 years; height, 171.7 ± 10.9 cm; body mass 69.6 ± 14.5 kg) completed in a randomised order (1) RE: 3 sets of 10 repetitions of 4 RE exercises at 60% of 1RM with paced breathing (2) REVM: repetition of the RE requirements with a VM during each of the last 6 repetitions of each set (3) VM: 12 sets of 6, 3 s VMs at a mouth pressure of 40 mm Hg. PWV was measured pre- and post-condition, with mean middle cerebral artery blood velocity, mean arterial blood pressure (MAP), heart rate, and partial pressure of end-tidal carbon dioxide measured during each visit.

**Results:**

An interaction effect for PWV (*P* = 0.024) was observed, with post hoc tests revealing increased PWV post REVM (*P* = 0.006, pre 5.4 ± 0.3 vs. post 6.1 ± 0.9 cm/s) and VM (*P* = 0.031, 5.7 ± 0.8 vs. 6.0 ± 1.0 cm/s), but not RE (*P* = 0.957, 5.7 ± 0.6 vs. 5.7 ± 0.8). REVM produced the greatest MAP increase from baseline (Δ37 ± 15 mm Hg, *P* < 0.05 vs. both RE and VM).

**Conclusion:**

These findings indicate that the VM contributes to the acute rise in post RE PWV when performed during RE and acutely increases PWV when performed repeatedly in isolation (Trial registration number ACTRN12624000876594, Date of registration 18/07/2024).

**Trial registration number:**

ACTRN12624000876594.

## Introduction

It is known that exercise confers many benefits to cardiovascular health (Sanchis-Gomar et al. 2022). However, exercise is a broad umbrella term that comprises different modalities that each produce profoundly different acute physiological responses and subsequent chronic adaptations. When considering the vascular adaptations to exercise, endurance/aerobic exercise increases central arterial compliance acutely following exercise (Saz-Lara et al. 2021), particularly when performed at higher intensities (Perissiou et al. 2018). At rest, regular aerobic exercise can increase carotid artery compliance, which reduces pulsatile blood velocity in the middle cerebral artery, indicative of a compensatory buffering mechanism to counteract the enhanced left ventricular systolic function associated with said exercise (Tomoto et al. 2015). These vascular adaptations also appear to extend to the cerebral circulation as indicated by increased middle cerebral artery compliance in young adults with increased cardiorespiratory fitness (Furby et al. 2019). However, the vascular adaptations to resistance exercise (RE) are less clear. It is purported that the repeated exposure to intermittent increases in blood pressure (BP) associated with habitual RE may increase arterial stiffness. Acutely, carotid artery compliance is reduced and beta stiffness increased immediately, and for 30 min following RE at 75% of the 1-repeition maximum (1RM), returning to baseline after 60 min (DeVan et al. 2005). At the same exercise intensity, Kingsley et al. (2017) reported an increase in augmentation index and aortic stiffness acutely following RE in resistance trained participants, with no observed sex differences. However, there is conflicting evidence regarding resting arterial stiffness following RE training. Miyachi et al. (2004) reported that following 4 months of RE training at 80% of 1RM, carotid compliance decreased by 19% and beta stiffness increased by 21%. However, Rakobowchuk et al. (2005) reported no change in beta stiffness or carotid cross-sectional compliance following 12 weeks of RE ≥ 80%. Meta-analyses of randomised controlled trials on resting pulse wave velocity (PWV) responses to RE interventions have revealed an intensity dependent effect, with Miyachi (2013) reporting that high intensity RE (> 70%1RM) increased arterial stiffness by ~ 12%, but moderate intensity RE (40–70%1RM) did not. More recent meta-analyses by Zhang et al. (2021) and Jurik et al. (2021), support this exercise intensity dependent effect, however, high intensity RE (> 70%1RM in Zhang et al. and > 80% in Jurik et al.) produced no change in PWV, with low-moderate intensity RE (30–70%1RM) reducing PWV (as indexed by carotid-femoral and brachial-ankle PWV), with the latter also supported by the analysis of Liu et al. (2023). Of note in the meta-regression analysis by Zhang, RE frequency, duration, sets or repetitions (i.e. volume) did not predict PWV outcomes, only intensity. Therefore, the intensity of the RE bouts appears to be central to the resting arterial stiffness adaptation to RE.

Importantly, in the studies and meta-analyses cited hitherto, the use of the Valsalva manoeuvre (VM) during RE is not explicitly discussed. During dynamic RE (RE that produces changes in muscle length, referred to herein as RE) sinusoidal perturbations in mean arterial blood pressure (MAP) occur, the magnitude of which are dependent on the recruited muscle mass, exercise intensity, body position, and the recruitment of the VM (Perry and Lucas 2021; MacDougall et al. 1985, 1992). The VM is a forced exhalation against a closed glottis and acts to increase spine stability and trunk rigidity during RE (Hackett and Chow 2013). The VM is unavoidable when force production exceeds ~ 80% of maximal voluntary contraction, or when fatigue is imminent during submaximal RE (MacDougall et al. 1992). Thus, when RE training at high intensities (e.g. targeting increases in muscular strength), or when performing RE to muscular fatigue, it is likely that the VM would be recruited. We have previously speculated that the VM may limit the increase in cerebral perfusion during the rapid rise in MAP associated with large muscle mass exercise (Perry et al. 2014c, 2020), but may also exacerbate the post-exercise hypotension and increase the likelihood of syncope (Compton et al. 1973). The VM may also underpin the vascular adaptations to regular RE. Heffernan et al. (2007b) reported that repeated VMs in isolation (60 total, in sets of 4) acutely increased central PWV, but pulsatile fluctuations in MAP of similar magnitude induced by RE alone (i.e. without the VM) did not. There is a paucity of data exploring the role of the VM in RE associated PWV responses with no studies to our knowledge reporting the impact of the VM on carotid-femoral PWV post RE. Mak and Lai (2015) reported an increase in carotid PWV (not carotid-femoral) of 0.24 m/s post RE with a VM, but the authors did not statistically compare this condition to the PWV data reported following RE exercise without the VM, and did not quantify the effect of the VM alone on PWV. Additionally, the training status of the participants was not reported, only bicep curls were used, VM intensity was not reported, and the recruitment of the VM was not confirmed (e.g. via BP responses). Thus, it is plausible that the intensity dependent effect of RE on arterial stiffness, acutely and following regular RE, may be due to VM utilisation at higher RE intensities.

Regular RE has many performance (e.g. muscular strength) and health related benefits. Whilst increased arterial stiffness is a strong and independent risk factor of premature coronary artery disease (Weber et al. 2004), RE can also lower blood pressure (Kelley and Kelley 2000) and is incorporated into the World Health Organisation physical activity guidelines. Thus, determining the factors that modulate RE associated cardiovascular system responses and adaptations are essential for safe RE performance and prescription, especially in untrained individuals wishing to begin RE. Thus, the aim of this study was to investigate the effect of RE with and without the VM on central arterial stiffness as indexed by pulse wave velocity. Additionally, we wished to explore the effect of the addition of the VM to the during RE haemodynamic responses. We hypothesised that (1) moderate intensity RE with a concomitant VM would acutely increase carotid-femoral PWV post RE, but RE alone would not (2) the VM alone would increase PWV (3) that the performance of the VM during RE would produce more pronounced perturbations in MAP compared to RE without the VM at the same exercise intensity (4) RE with a concomitant VM would increase MAP to a greater extent that the VM alone.

## Methods

### Ethics and informed consent

All participants were informed of the study procedures, aims, and risks of participation prior to providing written informed consent. This study was conducted in accordance with the latest revision of the declaration of Helsinki and was approved by the Central Health and Disability Ethics Committee (2024 EXP 19783) and was registered with the Australian New Zealand Clinical Trials Registry (ACTRN12624000876594).

### Participants

An a priori power analysis (G*Power v.3.1.9.4; Heinrich Heine University Düsseldorf, Düsseldorf, Germany) demonstrated that 13 participants were required based on conventional α (0.05) and β (0.80) values, and an effect size of 0.31 calculated from the central arterial PWV data (pre and post PWV data following isolated RE and VMs) of Heffernan et al. (2007b) as the primary dependent variable. A total of 15 participants (female = 8) were recruited for this study (mean ± SD: age, 24 ± 5 years; height, 171.7 ± 10.9 cm; body mass, 69.6 ± 14.5 kg; body mass index, 23.6 ± 4.6 kg m^2^). All participants were healthy, had no history of pulmonary, metabolic, cardiovascular, or neurological disease, were not taking any medication other than an intrauterine device (*n* = 1), and were non-smokers. Female participants self-reported their pregnancy status and menstrual cycle phase, with all visits occurring during the same menstrual phase (e.g. early follicular or luteal) despite evidence to indicate that the acute PWV responses to RE is not influenced by menstrual cycle phase (Augustine et al. 2018).

Participants had not completed regular RE (e.g. bodybuilding, powerlifting or weightlifting); defined as not having completed > 1 session per week for 3 or more consecutive weeks for the 6 months prior to study participation. Whilst 3 months of RE can increase cerebrovascular resistance (Thomas et al. 2021), this was extended to 6 months to account for potential individual variation. Furthermore, those that performed regular rowing exercise were excluded as it produces a similar haemodynamic profile to RE (Pott et al. 1997). Participants on average performed 1 ± 1 dedicated exercise session per week across the last 6 months prior to participating, lasting 64 ± 27 min (not including low intensity commuting either by foot or bicycle). A total of 9 participants self-reported the intensity of their exercise to be low, whilst 3 reported their exercise as moderate.

### Study design

Each participant visited the temperature-controlled laboratory on 4 occasions. The first occasion was to (1) confirm eligibility, which included the ability to insonate the M1 segment of the middle cerebral artery (MCA) as described below (2) to familiarise the participant with all study procedures and (3) teach the correct RE technique and estimate the 1 repetition maximum (1RM) for each of the selected experimental exercises—unilateral bicep curl (dominant arm), and bilateral leg press, chest press and leg extension. Unilateral bicep curl was selected to permit the concurrent non-invasive measurement of arterial BP via finger plethysmography on the non-dominant hand. Importantly, this unilateral bicep curl exercise at the loading described herein still produces sinusoidal fluctuations in BP (Korad et al. 2024b). Due to the untrained nature of the participants, to mitigate the risk of potential injury the 1RM was estimated using the equation described by Brzycki (1993): Weight ÷ (1.0278—(0.0278 × Number of repetitions)). From this, 60% of the estimated 1RM (60%1RM) was calculated as the working intensity for the subsequent visits (mean ± SD: Bicep curl predicted 1RM 20 ± 11 kg and 60%1RM 12 ± 3 kg; Leg press predicted 1RM 119 ± 46 kg and 60%1RM 71 ± 28 kg; Chest press predicted 1RM 43 ± 28 kg and 60%1RM 26 ± 17 kg; and leg extension 1RM 60 ± 31 kg and 60%1RM 36 ± 19 kg). All participants were able to complete all sets and repetitions.

### Experimental design

Visits 2, 3, and 4 were completed in a random order, these visits consisted of RE without VM (RE), RE with VMs (REVM), and VM only. Participants arrived at the laboratory having refrained from strenuous exercise and alcohol for 12 h, and caffeinated beverages for 4 h prior to testing. Prior to visit 2, participants were asked to record their diet and timing of food intake for 12 h prior to coming to the laboratory. Participants then repeated their recorded diet and the timing of food intake in all subsequent visits. Additionally, a list of foods with vasoactive potential (foods high in nitrates and anthocyanins) was provided to all participants so they could avoid their consumption for 12 h prior to all visits. Adherence was self-reported. Participants were instructed to stay hydrated the night before and the morning of visits 2, 3 and 4. Upon arrival, a urine sample was provided for the analysis of urine specific gravity (USG), and to confirm euhydration (USG < 1.020). The participant rested supine quietly for 5 min before the measurement of arterial BP and subsequently pulse wave velocity (PWV, pre-measures). Following the measurement of PWV participants sat in a chair for instrumentation and the measurement of additional baseline variables (e.g. MCAv). Following the completion of the visit requirements (see sections immediately below), the participant began 5 min of supine rest before the measurement of arterial BP and PWV (post-measures). The participant then sat upright in a chair for the remaining post-exercise measures (e.g. MCAv).

### Valsalva manoeuvre visit

During the VM visit, the participants performed 12 ‘sets’ of 6 VMs (72 total) at a mouth pressure of 40 mm Hg. Each VM lasted 3 s (s) and was separated by 2 s break (3 s “on”, 2 s “off”) as per Heffernan et al. (2007b), with verbal instructions given by the researchers. The 3 s VM, which was 1 s longer than in the REVM described below, enabled the participants to achieve a stable mouth pressure using visual real-time mouth pressure feedback displayed on a computer screen. The mouth pressure of 40 mm Hg was selected as this was the approximate peak mouth pressure produced when participants were able to perform a VM freely during RE (Perry et al. 2014c). Each VM set was separated by 3 min rest. During the VM visit all VMs were completed in the seated position.

### Resistance exercise visit

For the RE visit, participants performed 3 sets of 10 repetitions of a bicep curl, leg press, leg extension, and chest press at 60% of the estimated 1RM. The intensity of 60%1RM was selected to enable untrained participants to complete the RE at the required tempo without fatiguing, which is important as the VM as a lifter approaches fatigue (MacDougall et al. 1992). The order of the exercises was randomised, excluding the bicep curl which was completed first. Haemodynamic measurements were recorded only during the bicep curl which was unilateral to allow for the concurrent measurement of BP via finger photoplethysmography and to confirm the performance of the VM during the REVM visit (see Resistance Exercise with Valsalva Manoeuvre Visit heading below). Each repetition was timed to a metronome which provided both visual and audio feedback. Each repetition was 4 s in length, with 2 s for both concentric and eccentric phases. Additionally, exhalation was performed during the eccentric phase of each movement, whilst inhalation occurred during the concentric phase. The researchers provided verbal coaching of the ventilation pattern during all sets and exercises. Protocol viability was tested in untrained participants prior to commencing the study and practiced by each participant during the familiarisation session.

### Resistance exercise with Valsalva manoeuvre visit

**Fig. 1:**
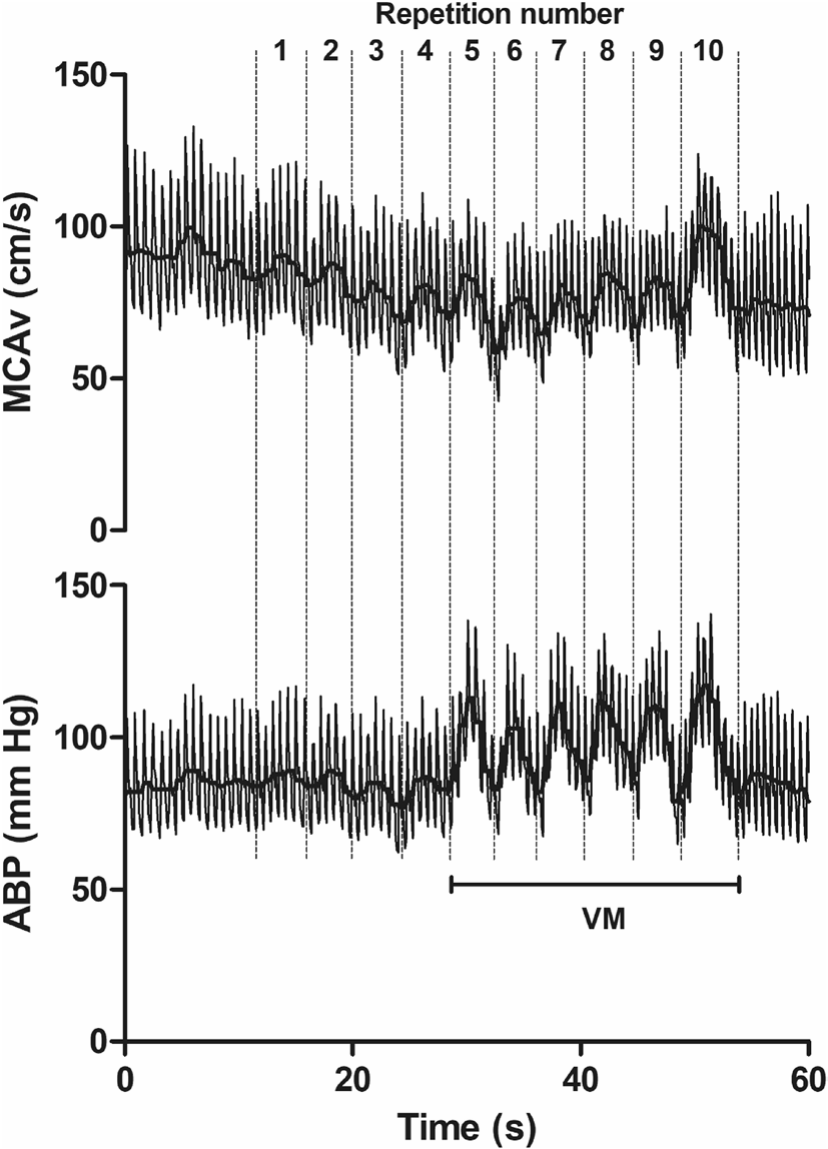
Middle cerebral artery blood velocity (MCAv) and arterial blood pressure (ABP) responses to 60%1RM unilateral bicep curl in one participant. The thick line in the MCAv and ABP traces represent the mean MCAv and mean arterial blood pressure respectively. The vertical dotted lines represent the timing of each repetition, with a Valsalva manoeuvre (VM) being performed in each of the last 6 repetitions of the set during the concentric phase only

During the REVM visit the exercise parameters (i.e. number of repetitions and sets, tempo, and exercises) were matched to the RE visit. However, a VM was performed during the concentric phase of each of the last 6 repetitions, in each set of RE for all exercises. For the first 4 repetitions of each set, the breathing pattern matched that of the RE visit. The total number of VMs (72) was matched with the VM visit (with the exception of the practice VMs described below) and the rest periods between each exercise set (3 min) matched both the VM and RE visits. Performing the VM in the last 6 repetitions was selected because 1) when lifting submaximal loads the VM can be utilised when approaching fatigue (i.e. later repetitions within a set) (MacDougall et al. 1992), and 2) pilot testing revealed that beginning the set with the ventilation of the RE visit enabled untrained participants to synchronise the repetition pacing and breathing timing before performing the VM. After baseline data were captured, participants practiced the VM in isolation with real-time mouth pressure feedback (as per the VM visit described above), the participants then repeated the VM without the mouthpiece and mouth pressure feedback. As the VM phase I BP response is intensity dependent (Perry et al. 2014b) the BP responses between the VMs in isolation with and without visual mouth pressure feedback were compared to confirm that the VM intensity was matched between visits. If the BP responses were different between VMs with and without visual feedback, the participant would repeat the aforementioned process until an appropriate BP response without visual feedback was achieved. The average number of practice VMs before initiating the bicep curl exercise was 6 ± 1. The performance of the VM during the bicep curl exercise of the REVM visit was confirmed in a similar manner (see Fig. 1).

### Systemic haemodynamics

Heart rate (HR) was measured using a three-lead electrocardiogram (ADInstruments, Australia). Non–invasive beat-to-beat measurement of BP was recorded by finger photoplethysmography (Human NIBP Nano System, ADInstruments). A cuff was placed on the middle phalanx of the middle finger and index finger on the non-dominant hand. The height correction unit was used to reference the level of the heart and checked against brachial BP measurements recorded via automated sphygmomanometer (Suresigns VM4, Philips Medical Systems, Philips, The Netherlands).

### Carotid-femoral pulse wave velocity (PWV)

Carotid-femoral PWV was measured using a high-fidelity tonometer (SphygmoCor CvMS, AtCor Medical), with electrocardiogram (3-lead). The participant rested for 5 min in a supine position. Following 5 min of rest, brachial BP measurements were recorded via automated sphygmomanometer and completed in duplicate. If systolic blood pressure (SBP) varied by > 5 mm Hg a third measurement was recorded and the last 2 measurements used. The last BP recorded was used for the calculation of PWV. The right common carotid and femoral pulses were palpated and marked. The distance between each marked pulse and the suprasternal notch was measured. The tonometer was placed over the common carotid artery first and then the femoral artery to obtain a waveform. Waveforms were continuously collected for 10 s, and measurements were taken in duplicate, and if the returned values differed by > 0.5 m/s, a third was completed. The quality of the recording was assessed using SphygmoCor CvMS software and measurements were deemed acceptable if the standard deviation was ≤ 10%. Measurements within each visit were completed by the same experienced operator. Pulse wave velocity was calculated as described by Butlin and Qasem (2017):

$$Carotid-femoral PWV=\frac{distance \, femoral-distance \, carotid}{Pulse \, transit \, time femoral-pulse \, transit \, time \, carotid}$$

where: PWV = pulse wave velocity, Distance femoral = distance from the palpated femoral pulse to the suprasternal notch, Distance carotid = distance from the palpated carotid pulse to the suprasternal notch, Pulse transit time femoral = transit time from the R wave of the electrocardiogram (ECG) to the foot of the applanated waveform obtained from the femoral artery, Pulse transit time carotid = transit time from the R wave of the ECG to the foot of the applanated waveform obtained from the carotid artery.

### Heart rate

Heart rate was measured during the seated rest periods (pre- and post-) and during the performance of RE and VM via three-lead ECG (ADInstruments).

### Middle cerebral artery blood velocity

Middle cerebral artery blood velocity (MCAv) was measured using transcranial Doppler Ultrasonography (Doppler-BoxX, DWL Compumedics). An adjustable headband was used with a 2 MHz ultrasound probe fixed in position over the temporal window, above the zygomatic arch. The M1 segment of the middle cerebral artery MCA was located using search techniques described elsewhere (Willie et al. 2011). Ultrasound gel was used between the probe and skin to ensure the highest quality image. It was not possible to adequately insonate the MCA bilaterally in all individuals and thus the MCAv and cerebrovascular data presented herein are from the MCA with the best quality signal. Importantly, we have previously shown that the MCAv responses in the contralateral and ipsilateral MCA to the exercising arm during bicep curls at 6–0% of 1RM are not different (Korad et al. 2024b).

### Partial pressure of end-tidal carbon dioxide

The partial pressure of end-tidal carbon dioxide (P_ET_CO_2_) was collected via a nasal cannula, and measured using a calibrated online gas analyser (ML206 Gas Analyser, ADInstruments, Australia) to account for the influence of arterial carbon dioxide content on cerebral blood flow. The gas analyser was calibrated to a specific known gas concentration before each visit.

### Urine analysis

Due to the finding that hydration status can influence cerebrovascular regulation (Perry et al. 2016), a handheld refractometer (Atago Co., Ltd, Tokyo, Japan) was used to determine hydration status before each visit. All participants were instructed to consume 500 mL of water the night before the visit and an hour before coming into the laboratory. If the participant was hypohydrated (indicated by a USG > 1.020), 500 mL of water was consumed and another urine sample was tested 30 min after water consumption, which was sufficient to produce a USG of < 1.020 in all instances where retesting was required. The mean USG across all visits was 1.0089 ± 0.0058.

### Data acquisition

All data were collected continuously using an analogue to digital converter (PowerLab, ADInstruments) interfaced with a computer, then analysed using LabChart software (v.8.1.28 ADInstruments).

### Data analysis

Mean MCAv (MCAv_mean_) and mean arterial blood pressure (MAP) were calculated using the mean velocity of the raw MCAv and ABP waveform respectively. The cerebrovascular conductance index (CVCi) was calculated using the formula CVCi = MCAv_mean_/MAP. Given RE produces sinusoidal fluctuations in MAP and MCAv, a simple average across the set does not reflect the sinusoidal nature of the BP profile (Perry and Lucas 2021). As the acute increases in MAP are purported to drive the potential changes in arterial stiffness during RE it is important to quantify the magnitude and direction of the produced changes in MAP as previously described (Korad et al. 2024a). Subsequently, cardiovascular and cerebrovascular variables at the MAP zenith and nadir values for each repetition of bicep curl exercise (RE and RE + VM), and for each VM for the first 3 sets of the VM visits, were identified and extracted. At each of these points the absolute change from the baseline immediately preceding the set was calculated and then averaged across all repetitions and sets (referenced henceforth as Δ zenith and Δ nadir). Additionally, the difference between the Δ zenith and Δ nadir (Δ difference) is reported to inform the magnitude of said fluctuations in variables of interest.

### Statistical analysis

All data were analysed using SPSS statistical software v.28 (IBM Corp., Armonk, NY, USA). PWV, MAP, SBP and DBP pre- and post-visit were analysed by two-way repeated measures analysis of variance (ANOVA, visit x time, 2 × 3—where time refers to the pre-and post-timepoints). The Δ zenith, Δ nadir and Δ difference were analysed using a one-way ANOVA. For the analysis of the REVM visit data only the last 6 repetitions where a VM was utilised were included in this analysis. However, to confirm the effect of the VM during the REVM visit the pooled mean Δ zenith, Δ nadir and Δ difference across the first 4 repetitions (without a VM) of all sets were compared to the pooled mean of the last 6 repetitions (with a VM) across all sets (see Fig. 1) using paired t-tests. For the VM visit, only the first 3 sets were used which time align with the RE and REVM visits. Baseline (pre) variables across all visits were compared using a one-way ANOVA. The level of statistical significance was set a priori at *P* ≤ 0.05. When a significant interaction effect was observed, differences between pre- and post- dependent variables were isolated using post hoc pairwise comparisons with Bonferroni correction where necessary. All data are displayed as mean ± standard deviation (SD).

## Results

Initial baseline data (pre intervention) for all variables were not different between visits (pooled means ± SD: PWV, 5.6 ± 0.7 cm/s (*P* = 0.328); MAP, 83 ± 7 mm Hg (*P* = 0.639); SBP, 112 ± 9 (*P* = 0.767); DBP, 69 ± 6 (*P* = 0.631); MCAv_mean_, 71 ± 12 cm/s (*P* = 0.369); P_ET_CO_2_, 39 ± 4 mm Hg (*P* = 0.447); HR, 85 ± 10 bpm (*P* = 0.366).

The performance of the VM during the last 6 repetitions of the bicep curl exercise during the REVM visit was confirmed by the greater MAP Δ zenith (first 4 repetitions = 9 ± 7 mm Hg vs. last 6 repetitions = 37 ± 15 mm Hg, *P* < 0.001) and Δ difference (7 ± 2 mm Hg vs. 34 ± 14 mm Hg, *P* < 0.001), with greater increases in HR at Δ zenith (10 ± 9 bpm vs. 23 ± 15 bpm, *P* = 0.001).

**Fig. 2:**
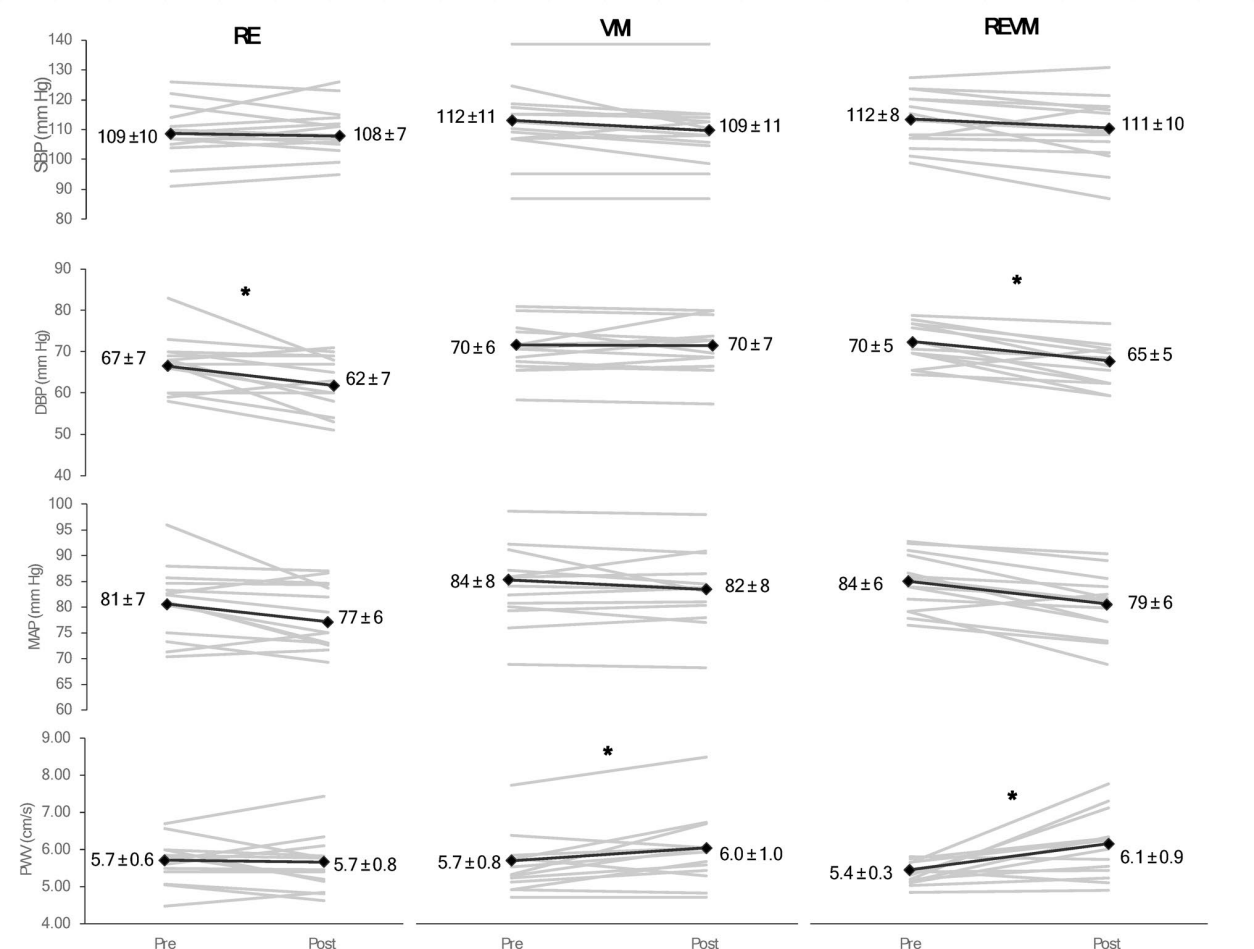
Individual resting (supine) pre- and post-systolic arterial blood pressure (SBP), diastolic arterial blood pressure (DBP), mean arterial blood pressure (MAP) and pulse wave velocity (PWV) pre- and post- resistance exercise (RE), Valsalva manoeuvre (VM), and resistance exercise with a Valsalva manoeuvre (REVM). The solid diamonds and bold black lines represent the mean group plot for each variable, with the values adjacent to this plot representing the group mean value and standard deviation. *Statistically different from within visit pre- value (*P* < 0.031). *n* = 14 for PWV and *n* = 15 for BP data

The individual and mean pre- and post- intervention data for PWV, MAP, SBP and DBP are reported in Fig. 2. For one participant an adequate PWV measurement was unable to be obtained, and therefore *n* = 14 for the PWV measures. A significant interaction effect for PWV (*P* = 0.024) was observed, with post hoc tests revealing an increase in PWV post REVM (*P* = 0.006) and VM (*P* = 0.031), but not for RE (*P* = 0.957, see Fig. 2 for values). Analysis of DBP revealed an interaction effect (*P* = 0.019), with a significant reduction in DBP post RE (*P* = 0.023) and REVM (*P* = 0.001), but not for VM (*P* = 0.943, see Fig. 2 for values). A main effect of time was apparent for both SBP (*P* = 0.050) and MAP (*P* = 0.002), with no other main or interaction effects achieving significance (all *P* > 0.070, see Fig. 2 for values).

Due to the BP recording being unacceptable in 5 participants, and the zenith and nadir values being identified from the BP waveform, the Δ zenith, Δ nadir, and Δ difference data for MCAv_mean,_ MAP, SBP, DBP, CVCi and HR are reported for *n* = 10 in Table 1. Increases from the preceding baseline (*Δ* zenith) for systolic MCAv during REVM (8 ± 2 cm/s) and VM (4 ± 2 cm/s) were greater compared to RE (-7 ± 4 cm/s, post hoc test vs. REVM, *P* = 0.003 and vs. VM, *P* = 0.028). REVM also increased MAP (post hoc test vs. RE *P* < 0.001 and vs. VM *P* = 0.050, see Table 1 for ANOVA *P* values) and HR (vs. RE *P* = 0.006 and vs. VM *P* = 0.004) at *Δ* zenith. However, the absolute reduction in MAP at *Δ* nadir was greatest for VM and was significantly lower than both RE (post hoc test *P* < 0.001) and REVM (*P* = 0.001), although HR for REVM was greatest (vs. RE *P* = 0.013 and vs. VM *P* = 0.007). For Δ difference, REVM increased systolic MCAv perturbations relative to RE (17 ± 9 vs. 5 ± 3 cm/s, post hoc test *P* = 0.003), however VM (13 ± 9 cm/s) was not different from REVM or RE (both *P* > 0.07). For *Δ* difference there was no difference between REVM and VM for all variables (all *P* > 0.05), although both VM and REVM showed greater perturbations compared to RE for MAP, SBP, DBP and MCAv_mean_ (for all VM vs. RE *P* < 0.001 and REVM vs. RE *P* < 0.001). Given the performance of the VM during the visit requirements for VM and REVM, use of P_ET_CO_2_ as a proxy for arterial carbon dioxide content is limited. However, as breathing was paced for the RE visit, P_ET_CO_2_ data could be collected, and on average the absolute change in P_ET_CO_2_ from the immediately preceding baseline was -5 ± 2 mm Hg.

**Table 1:** Change from baseline at zenith, nadir, and the difference between zenith and nadir during each repetition/Valsalva manoeuvre

	RE	VM	REVM	*P* values
*Δ zenith*				
MCAv_mean_ (cm/s)	-5 ± 5	3 ± 7	4 ± 11	0.051
MAP (mm Hg)	14 ± 8	24 ± 12	37 ± 15*†	< 0.001
SBP (mm Hg)	16 ± 10	27 ± 16	41 ± 17*	0.003
DBP (mm Hg)	11 ± 8	21 ± 12	32 ± 13*	0.001
CVCi (cm/s/mm Hg)	-0.1 ± 0.0	-0.1 ± 0.1	-0.2 ± 0.1	0.124
HR (bpm)	7 ± 8	6 ± 8	23 ± 15*†	0.002
*Δ nadir*				
MCAv_mean_	-11 ± 5	-18 ± 8	-14 ± 6	0.080
MAP	6 ± 8	-9 ± 4*‡	3 ± 8	< 0.001
SBP	8 ± 9	-10 ± 7*‡	3 ± 15	0.003
DBP	7 ± 7	-4 ± 3*‡	6 ± 5	< 0.001
CVCi	-0.1 ± 0.0	-0.1 ± 0.1	-0.2 ± 0.1	0.433
HR	6 ± 11	5 ± 6	21 ± 15*†	0.004
*Δ difference*				
MCAv_mean_	7 ± 3	20 ± 5*	18 ± 6*	< 0.001
MAP	7 ± 2	33 ± 14*	34 ± 14*	< 0.001
SBP	9 ± 4	37 ± 15*	37 ± 18*	< 0.001
DBP	5 ± 2	25 ± 12	26 ± 10	< 0.001
CVCi	0.0 ± 0.0	0.0 ± 0.1	0.0 ± 0.1	0.176
HR	1 ± 3	2 ± 4	2 ± 4	0.882

Data are averaged across all repetitions and sets

Data are mean ± SD, *n* = *10*

*REVM* resistance exercise with a Valsalva manoeuvre, *RE* resistance exercise, *VM* Valsalva manoeuvre, *MCAv*_*mean*_ Middle cerebral artery blood velocity mean, *MAP* Mean arterial blood pressure, *DBP* Diastolic arterial blood pressure *SBP*, systolic arterial blood pressure, *CVCi* Cerebrovascular conductance index, *HR* Heart rate

*Different from RE, P < 0.05

†Different from VM, P < 0.05

‡Different from REVM P < 0.05

Note that 1) the data presented for REVM refers to the mean of the last 6 repetitions (where a VM was performed see figure (1), and (2) the data for VM are presented as the mean across the first 3 sets to time align with REVM and RE

## Discussion

To our knowledge, this is the first study to compare the PWV responses between RE, the VM alone, and RE with a VM (REVM). The key findings of the study are that 1) PWV acutely increased post REVM and VM, but not following RE, which confirmed our first and second hypotheses 2) Peak MAP, as indexed by *Δ* zenith, was greater during REVM than RE and VM. However, the size of the perturbations in MAP (*Δ* difference) were similar between REVM and VM, and both were greater than RE. These data confirm our third and fourth hypotheses (3) The VM and REVM produced greater perturbations in MCAv_mean_ compared to RE (4) REVM and RE both acutely decreased DBP. Collectively, these data indicate that when RE intensity is matched, the addition of the VM during RE (REVM) produces much more profound perturbations in BP and MCAv_mean_ during exercise, and acutely increases PWV immediately following RE. Thus, the performance of a VM is an important consideration when quantifying the acute cardio- and cerebrovascular responses to RE.

We report an increase in carotid-femoral PWV immediately following REVM (see Fig. 2) and confirm the notion that the VM contributes to the post RE PWV increase. As the VM is typically utilised when force production exceeds ~ 80% of maximal voluntary contraction, or when approaching fatigue at submaximal loads (MacDougall et al. 1992), it is possible that the participants within studies investigating the vascular responses to fatiguing or high intensity RE would be inadvertently utilising the VM. Indeed, studies employing moderate to high intensity, or fatiguing RE have reported reduced arterial compliance/increased stiffness acutely following RE (DeVan et al. 2005; Heffernan et al. 2007a; Lefferts et al. 2014, 2015). However, the utilisation of the VM in these studies is not reported or discussed. The increase in PWV acutely following RE is supported by two systematic reviews and meta-analyses by Pierce et al. (2018) and Jurik et al. (2021), although across both meta-analyses the performance of the VM was only mentioned in one study, that of Mak and Lai (2015). In addition to the lack of reporting on VM status, differences in exercise parameters (exercise selection, intensities, volume, and rest periods) and training status of the participants, mean direct comparisons between studies is challenging.

Whilst the effects of the VM are clear here in the acute context, how these data inform chronic adaptations requires further investigation. Data from cross sectional studies indicate that strength/resistance trained individuals demonstrate increased arterial stiffness relative to controls (Bertovic et al. 1999; Miyachi et al. 2003; Kawano et al. 2008), and interventional studies using high intensity RE interventions also report a reduction in arterial compliance (Miyachi et al. 2004), although the latter is not a consistent finding (Rakobowchuk et al. 2005). Multiple meta-analyses of randomised controlled trials have reported an intensity dependent effect of RE, with higher intensities generating increased arterial stiffness (Miyachi 2013), whilst lower intensity RE (e.g. < 70%1RM) may reduce arterial stiffness (Zhang et al. 2021; Jurik et al. 2021). Similarly to the acute studies, the utilisation of the VM is not discussed, and it again raises the possibility that the intensity dependent effect of RE on resting PWV may be influenced by the utilisation of the VM. Given the findings in the current study, further investigation is required to elucidate the underlying mechanisms of intensity dependent nature of the resting arterial stiffness/compliance adaptations to regular RE training. Transparency around the use of the VM in both acute, and training-based studies, is required to help reconcile the differences between studies.

These data also indicate a significant increase in PWV following repeated VMs and support the findings of Heffernan et al. (2007b). Of note, Heffernan et al. (2007b) reported a ~ 0.7 m/s increase in central PWV, which is more than double the increase in PWV reported here. Despite similar timing and duration of the VM to the current study, Heffernan et al. (2007b) used a mouth pressure of 50 mmHg to produce a target rise in SBP of 45–55 mm Hg to match the rise in SBP achieved during the RE employed (unilateral leg press and leg extension exercise at 75% of 1RM). Of note, that despite the similar BP perturbations between RE and VM conditions, only repeated VMs produced an increase in PWV. In the current study we used a mouth pressure of 40 mmHg, and report a smaller rise in MAP (*Δ* zenith) during VM compared to REVM, yet both the REVM and VM increased PWV. Although BP was measured during unilateral bicep curl exercise, we also employed bilateral large muscle mass RE (e.g. leg press) that would produce larger perturbations in BP than are reported in Table 1, even without a concurrent VM (e.g. as in Edwards et al. (2002)). Thus, although speculative, the PWV response may not be determined by the peak BP response. One possible explanation is that sympathetic nervous system activity is elevated by the performance of the VM. The VM alone can be used to assess autonomic function (Novak 2011) as the BP perturbations generate phase dependent autonomic responses. That is, a sympathetically mediated increase in HR during phase IIb (Tiecks et al. 1995), and potentially sympathetically mediated cerebral vasoconstriction during phase IV (Zhang et al. 2004). There is growing evidence that sympathetic nervous system activity may modulate arterial stiffness (Nardone et al. 2020) with elevated sympathetic nervous system activity increasing PWV (Swierblewska et al. 2010; Holwerda et al. 2019). Cross-sectional data from RE trained individuals indicates a higher arterial stiffness relative to aerobically trained individuals that was associated with greater muscle sympathetic nervous system activity (Smith et al. 2015). However, as sympathetic nervous system activity was not measured in the current study this hypothesis requires further exploration.

Increased PWV is an independent predictor of cardiovascular mortality (Xuereb et al. 2023). Stiffer central arteries (e.g. aorta) increase systolic pressure augmentation and end organ pulsatile pressure in low pressure vascular beds of end organs such as the brain, which mediates microvascular damage (Chirinos et al. 2019). Indeed, increased PWV may be a modifiable risk factor for cognitive impairment and dementia (Pase et al. 2016). However, RE produces a plethora of benefits, including that to the cardiovascular system, as evidenced by an acute reduction in DBP in the current study. The reduction in DBP following RE, that persists with the performance of the VM, is in agreement with the findings of others. A meta-analysis of the BP responses to a single bout of RE reveals a lower BP in both normotensive and hypertensive patients post RE for up to 24 h, with the effect more pronounced following large muscle mass RE (Casonatto et al. 2016). Regular progressive RE training also yields a significant lowering effect on resting MAP, SBP, and DBP in normotensive adults (Kelley and Kelley 2000). The results of the current study therefore suggest that despite the PWV response, the use of the VM may have no impact on the BP lowering effect of RE.

The current data indicate that there were significantly greater perturbations (*Δ* difference) in MCAv_mean_ during REVM and VM compared to RE. We have previously reported no change in peak MCAv_mean_ between exercise intensities, despite MAP being significantly greater at the highest intensity RE (90% of 6RM) where a VM was utilised (Perry et al. 2014c). The most likely reason for this discrepancy in data sets is the paced breathing protocol used herein. As we were unable to measure P_ET_CO_2_ during the REVM and VM, inferences can only be made by the P_ET_CO_2_ during RE, where there was an average 5 mm Hg reduction. The subsequent hypocapnia induced by the paced breathing during RE likely underpins the reduction in MCAv_mean_, as evidenced by MCAv_mean_ remaining below baseline at *Δ* zenith. Romero and Cooke (2007) previously reported that pre-leg press RE hyperventilation to an end tidal CO_2_ of 3 and 2% decreases within exercise mean MCAv_mean_ (averaged across the entire set) by 14 and 25%, respectively, whilst MCAv_mean_ increased by 12% without pre-RE hyperventilation. Similarly, if P_ET_CO_2_ is clamped to baseline levels during handgrip exercise MCAv_mean_ is elevated compared to baseline and is greater than the same exercise with permitted fluctuations in P_ET_CO_2_ (Braz et al. 2014). Thus, hyperventilation and the subsequent reduction in arterial CO_2_ content underpins the reduction in MCAv_mean_ despite the perturbations in MAP, and ostensibly cerebral perfusion pressure. During VM and REVM the MCAv_mean_ profile reflects the greater perturbations in MAP (e.g. in Fig. 1). The high pass filter nature of the cerebral circulation is exemplified during dynamic RE, where the sinusoidal fluctuations in MAP typically occur at a frequency where autoregulatory processes are less effective. In the current study the oscillatory frequency of MAP is ~ 0.25 Hz, which exceeds the proposed upper frequency limit for cerebral autoregulation (Panerai et al. 2023; Burma et al. 2024). As these perturbations are occurring too quickly for cerebral autoregulation to effectively counter, more pronounced perturbations in MAP produced by the VM are translated to the cerebral circulation and produce concurrent changes in MCAv_mean_. When MAP is already rapidly changing due to the background of large muscle mass RE (e.g. leg press RE), or when RE includes an orthostatic component (e.g. upright squatting as in Perry et al. (2014c)) the effect of the VM on the MCAv_mean_ response may diminish. During small muscle mass RE the VM dominates the BP profile (bicep curl exercise as in Fig. 1) and subsequently perturbs MCAv_mean_ similar to isometric RE (Pott et al. 2003; Perry et al. 2020). However, as the current study utilised transcranial Doppler to measure MCAv, and not blood flow, further research is required to confirm these hypotheses.

### Perspectives and future directions

To discern all phases of the VM, a straining period of > 10 s is required (Perry et al. 2014a). As the VM duration used herein was short (i.e. 2 s during REVM), whether these findings persist with longer duration VMs is unknown. However, the following challenges exist when considering the effect of VM duration on the PWV responses in during concurrent RE (1) the VM is typically recruited in the concentric phase of dynamic RE, to discern all VM phases would potentially require a contraction of > 10 s; (2) VM phases are visible during isometric exercise (e.g. 20 s bilateral leg extension as in Perry et al. (2020)) with concurrent VM (40 mm Hg). Although whether all VM phases would be visible during dynamic RE is unclear as the RE itself could induce a rapidly changing MAP, especially RE that utilises a large muscle mass with an orthostatic component (e.g. squatting); (3) point 2 may also be confounded by a single VM spanning multiple repetitions; (4) the body position also impacts the BP responses during the VM, as standing reduces the BP throughout the straining period (phases I though IIb) relative to supine posture (Pott et al. 2000). The RE performed herein were in the seated or semi-recumbent positions, and performing the VM during standing RE may exacerbate the BP perturbations 5) More intense VMs also produce more pronounced perturbations in BP, including greater increases during phase I and decreases during phase III (Perry et al. 2014a). It is possible that participants may self-select a VM intensity during RE and clamping VM intensity may not be ecologically valid. Further research is required to explore the VM parameters and how these may impact the PWV responses during RE.

The World Health Organisation physical activity guidelines for adults include the performance of muscle-strengthening exercise (e.g. RE) for ≥ 2 day per week at a moderate or greater intensity (Bull et al. 2020). Thus, it is important to understand the physiological adaptations to RE to best inform safe practice for those wishing to engage in this beneficial form of exercise. As such, the current research utilised untrained participants that were not regularly completing RE. We have previously shown differential BP responses during and following RE between RE trained and untrained participants. That is, greater BP responses during RE (Korad et al. 2024a), with greater reductions in MAP upon standing after leg extension RE (Korad et al. 2025). Further research is required to ascertain if habitually RE-trained participants, particularly those that train at high intensities and utilise the VM (e.g. strength athletes), exhibit similar within RE haemodynamics and acute PWV responses to RE.

Combined exercise (RE and aerobic/endurance) also requires consideration as a mechanism to offset potential detrimental impacts of RE on cardiovascular function. Using a randomised controlled design, Kawano et al. (2006) reported a ~ 20% reduction in carotid arterial compliance following 4 months of moderate intensity RE, but no change in the group that performed intensity matched RE with regular aerobic cycling exercise. Combined exercise training has been shown to reduce PWV in the general population and in some clinical populations as shown in a systematic review of systematic reviews by Liu et al. (2023). Liu et al. (2023). reported that a reduction in PWV in in those with hypertension and cardiovascular disease, but no change for those with type II diabetes mellitus. Thus, combined exercise could be used to exploit the modality dependent adaptations to exercise and offset the increased PWV/arterial stiffness produced by high intensity RE with a VM.

### Limitations

Whilst care was taken to match the VM intensity between REVM and VM by observing the BP responses, the actual VM intensity, and thus overall VM “dose” during REVM is not known and is a limitation of the current study. Measurement of oesophageal pressure throughout the RE and REVM bouts should be considered in future studies to accurately exclude VM use during RE and quantify VM intensity during REVM. Nevertheless, when VM intensity was controlled when visual feedback was available in the VM visit, PWV was also increased. Further research is required to assess the effect of manipulating VM parameters (e.g., intensity and duration) during RE on the during and post-RE vascular responses. Similarly, the results herein are limited to the exercise parameters used (60% of 1RM), and to a healthy young cohort. We also only measured PWV immediately (~ 5 min) following the REVM and VM conditions, thus the time course of the elevation and subsequent decay in PWV is unknown. However, this was by design, as this study provides proof of concept that the VM does indeed influence the arterial response to RE acutely, and future research should investigate the role of the VM in the PWV response to RE using randomised controlled trial training studies using a larger sample size. Exploration of the mechanisms of action, particularly the involvement of the autonomic nervous system, also requires attention.

The current study used transcranial Doppler (TCD) to assess MCAv_mean_ as a proxy for cerebral blood flow. However, for TCD derived MCAv_mean_ to accurately reflect blood flow in the MCA a stable arterial diameter is needed. The stability of large artery diameter in the brain has recently been challenged by MRI studies that have indicated: (1) sympathetically mediated vasoconstriction of the MCA during handgrip exercise (Verbree et al. 2017) (2) a non-linear change in MCA diameter, with variations in P_ET_CO_2_ ranging between ~ -7.5 to ~  + 7.5 mm Hg from normocapnia not altering MCA diameter, but greater hypercapnia (i.e. 15 mm Hg increase from normocapnia) producing dilation of the MCA. Given the change in respiration caused by the VM in two of the visits, P_ET_CO_2_ cannot be used as an accurate representation of arterial CO_2_, and the arterial CO_2_ response to VM and REVM cannot be determined from the current data and confounds data interpretation. As the actual diameter of the MCA cannot be confirmed, the cerebrovascular derived metrics in the current study must be interpreted with caution.

## Conclusion

The findings of the current study indicate that the VM contributes to the acute rise in PWV when performed in conjunction with RE and acutely increases PWV when performed repeatedly in isolation. Utilisation of the VM during RE also increases the magnitude of the rise, and size of the fluctuations, in MAP during each repetition compared to RE with paced breathing. These more profound perturbations in MAP during RE produce by the VM are translated to the cerebral circulation such that the MCAv response replicates that of MAP. However, DBP was acutely reduced following RE with and without a concurrent VM. The collective effect of these responses requires further investigation to determine the chronic effect of an increased arterial stiffness, particularly when RE may lower BP even when a VM is utilised. Given the profound influence of the VM on the cardio- and cerebrovascular responses to RE, the performance of the VM should be disclosed and discussed in papers within this topic area, particularly at high RE intensities where the VM is more likely to be performed involuntarily.

## Data Availability

The datasets generated during the current study are available from the corresponding author upon reasonable request via email.

## Abbreviations

ABP: Arterial blood pressure
ANOVA: Analysis of variance
BP: Blood pressure
CVCi: Cerebrovascular conductance index
DBP: Diastolic blood pressure
ECG: Electrocardiogram
HR: Heart rate
MAP: Mean arterial blood pressure
MCA: Middle cerebral artery
MCAv: Middle cerebral artery blood velocity
MCAv_mean_: Mean middle cerebral artery blood velocity
P_ET_CO_2_: Partial pressure of end-tidal carbon dioxide
PWV: Pulse wave velocity
RE: Resistance exercise
REVM: Resistance exercise with a Valsalva manoeuvre
SBP: Systolic blood pressure
USG: Urine specific gravity
VM: Valsalva manoeuvre
1RM: One repetition maximum

## References

[CR1] Augustine JA, Nunemacher KN, Heffernan KS (2018) Menstrual phase and the vascular response to acute resistance exercise. Eur J Appl Physiol 118(5):937–946 29455431 10.1007/s00421-018-3815-1

[CR2] Bertovic DA, Waddell TK, Gatzka CD, Cameron JD, Dart AM, Kingwell BA (1999) Muscular strength training is associated with low arterial compliance and high pulse pressure. Hypertension 33(6):1385–1391 10373221 10.1161/01.hyp.33.6.1385

[CR3] Braz ID, Scott C, Simpson LL, Springham EL, Tan BW, Balanos GM, Fisher JP (2014) Influence of muscle metaboreceptor stimulation on middle cerebral artery blood velocity in humans. Exp Physiol 99(11):1478–1487 25217497 10.1113/expphysiol.2014.081687

[CR4] Brzycki M (1993) Strength testing—predicting a one-rep max from reps-to-fatigue. J Phys Educ Recreat Dance 64(1):88–90. 10.1080/07303084.1993.10606684

[CR5] Bull FC, Al-Ansari SS, Biddle S, Borodulin K, Buman MP, Cardon G, Carty C, Chaput J-P, Chastin S, Chou R (2020) World Health Organization 2020 guidelines on physical activity and sedentary behaviour. Br J Sports Med 54(24):1451–1462 33239350 10.1136/bjsports-2020-102955 PMC7719906

[CR6] Burma JS, Neill MG, Fletcher EK, Dennett BE, Johnson NE, Javra R, Griffiths JK, Smirl JD (2024) Examining the upper frequency limit of dynamic cerebral autoregulation: considerations across the cardiac cycle during eucapnia. Exp Physiol 109(12):2100–2121 39382938 10.1113/EP091719 PMC11607623

[CR7] Butlin M, Qasem A (2017) Large artery stiffness assessment using SphygmoCor technology. Pulse 4(4):180–192 28229053 10.1159/000452448 PMC5290450

[CR8] Casonatto J, Goessler KF, Cornelissen VA, Cardoso JR, Polito MD (2016) The blood pressure-lowering effect of a single bout of resistance exercise: a systematic review and meta-analysis of randomised controlled trials. Eur J Prev Cardiol 23(16):1700–1714 27512052 10.1177/2047487316664147

[CR9] Chirinos JA, Segers P, Hughes T, Townsend R (2019) Large-artery stiffness in health and disease: JACC state-of-the-art review. J Am Coll Cardiol 74(9):1237–1263 31466622 10.1016/j.jacc.2019.07.012 PMC6719727

[CR10] Compton D, Hill P, Sinclair J (1973) Weight-lifters’ blackout. Lancet 302(7840):1234–1237 10.1016/s0140-6736(73)90974-4 4128562

[CR11] DeVan AE, Anton MM, Cook JN, Neidre DB, Cortez-Cooper MY, Tanaka H (2005) Acute effects of resistance exercise on arterial compliance. J Appl Physiol 98(6):2287–2291 15718412 10.1152/japplphysiol.00002.2005

[CR12] Edwards M, Martin DH, Hughson RL (2002) Cerebral hemodynamics and resistance exercise. Med Sci Sports Exerc 34(7):1207–1211 12131264 10.1097/00005768-200207000-00024

[CR13] Furby HV, Warnert EA, Marley CJ, Bailey DM, Wise RG (2019) Cardiorespiratory fitness is associated with increased middle cerebral arterial compliance and decreased cerebral blood flow in young healthy adults: a pulsed ASL MRI study. J Cereb Blood Flow Metab. 10.1177/0271678x19865449 31564194 10.1177/0271678X19865449 PMC7446564

[CR14] Hackett DA, Chow C-M (2013) The valsalva maneuver: its effect on intra-abdominal pressure and safety issues during resistance exercise. J Strength Cond Res 27(8):2338–2345 23222073 10.1519/JSC.0b013e31827de07d

[CR15] Heffernan KS, Collier S, Kelly E, Jae S, Fernhall B (2007a) Arterial stiffness and baroreflex sensitivity following bouts of aerobic and resistance exercise. Int J Sports Med 28(03):197–203 17024636 10.1055/s-2006-924290

[CR16] Heffernan KS, Jae SY, Edwards DG, Kelly EE, Fernhall B (2007b) Arterial stiffness following repeated Valsalva maneuvers and resistance exercise in young men. Appl Physiol Nutr Metab 32(2):257–264 17486167 10.1139/h06-107

[CR17] Holwerda SW, Luehrs RE, DuBose L, Collins MT, Wooldridge NA, Stroud AK, Fadel PJ, Abboud FM, Pierce GL (2019) Elevated muscle sympathetic nerve activity contributes to central artery stiffness in young and middle-age/older adults. Hypertension 73(5):1025–1035 30905199 10.1161/HYPERTENSIONAHA.118.12462 PMC6937199

[CR18] Jurik R, Żebrowska A, Stastny P (2021) Effect of an acute resistance training bout and long-term resistance training program on arterial stiffness: a systematic review and meta-analysis. J Clin Med 10(16):3492 34441788 10.3390/jcm10163492 PMC8397161

[CR19] Kawano H, Tanaka H, Miyachi M (2006) Resistance training and arterial compliance: keeping the benefits while minimizing the stiffening. J Hypertens 24(9):1753–1759 16915024 10.1097/01.hjh.0000242399.60838.14

[CR20] Kawano H, Tanimoto M, Yamamoto K, Sanada K, Gando Y, Tabata I, Higuchi M, Miyachi M (2008) Resistance training in men is associated with increased arterial stiffness and blood pressure but does not adversely affect endothelial function as measured by arterial reactivity to the cold pressor test. Exp Physiol 93(2):296–302 17911355 10.1113/expphysiol.2007.039867

[CR21] Kelley GA, Kelley KS (2000) Progressive resistance exercise and resting blood pressure: a meta-analysis of randomized controlled trials. Hypertension 35(3):838–843 10720604 10.1161/01.hyp.35.3.838

[CR22] Kingsley JD, Tai YL, Mayo X, Glasgow A, Marshall E (2017) Free-weight resistance exercise on pulse wave reflection and arterial stiffness between sexes in young, resistance-trained adults. Eur J Sport Sci 17(8):1056–1064 28671855 10.1080/17461391.2017.1342275

[CR23] Korad S, Mündel T, Perry BG (2024a) The effects of habitual resistance exercise training on cerebrovascular responses to lower body dynamic resistance exercise: a cross-sectional study. Exp Physiol 109(9):1478–1491 38888986 10.1113/EP091707 PMC11363110

[CR24] Korad S, Mündel T, Perry BG (2024b) Neurovascular coupling during dynamic upper body resistance exercise in healthy individuals. Exp Physiol. 10.1113/EP091970 39320059 10.1113/EP091970 PMC11607614

[CR25] Korad S, Mündel T, Perry BG (2025) Larger reductions in blood pressure during post-exercise standing, but not middle cerebral artery blood velocity, in resistance-trained versus untrained individuals. Exp Physiol 110(3):424–437 39721042 10.1113/EP092327 PMC11868030

[CR26] Lefferts WK, Augustine JA, Heffernan KS (2014) Effect of acute resistance exercise on carotid artery stiffness and cerebral blood flow pulsatility. Front Physiol 5:101 24678301 10.3389/fphys.2014.00101 PMC3958641

[CR27] Lefferts W, Hughes W, Heffernan KS (2015) Effect of acute high-intensity resistance exercise on optic nerve sheath diameter and ophthalmic artery blood flow pulsatility. J Hum Hypertens 29(12):744–748 25739332 10.1038/jhh.2015.12

[CR28] Liu H, Shivgulam ME, Schwartz BD, Kimmerly DS, O’Brien MW (2023) Impact of exercise training on pulse wave velocity in healthy and clinical populations: a systematic review of systematic reviews. Am J Physiol Heart Circ Physiol 325(5):H933–H948 37594481 10.1152/ajpheart.00249.2023

[CR29] MacDougall J, Tuxen D, Sale D, Moroz J, Sutton J (1985) Arterial blood pressure response to heavy resistance exercise. J Appl Physiol 58(3):785–790 3980383 10.1152/jappl.1985.58.3.785

[CR30] MacDougall J, McKelvie R, Moroz D, Sale D, McCartney N, Buick F (1992) Factors affecting blood pressure during heavy weight lifting and static contractions. J Appl Physiol 73(4):1590–1597 1447109 10.1152/jappl.1992.73.4.1590

[CR31] Mak WYV, Lai WKC (2015) Acute effect on arterial stiffness after performing resistance exercise by using the valsalva manoeuvre during exertion. Biomed Res Int 2015(1):343916 26539481 10.1155/2015/343916 PMC4619793

[CR32] Miyachi M (2013) Effects of resistance training on arterial stiffness: a meta-analysis. Br J Sports Med 47(6):393–396 22267567 10.1136/bjsports-2012-090488

[CR33] Miyachi M, Donato AJ, Yamamoto K, Takahashi K, Gates PE, Moreau KL, Tanaka H (2003) Greater age-related reductions in central arterial compliance in resistance-trained men. Hypertension 41(1):130–135 12511542 10.1161/01.hyp.0000047649.62181.88

[CR34] Miyachi M, Kawano H, Sugawara J, Takahashi K, Hayashi K, Yamazaki K, Tabata I, Tanaka H (2004) Unfavorable effects of resistance training on central arterial compliance a randomized intervention study. Circulation 110(18):2858–2863 15492301 10.1161/01.CIR.0000146380.08401.99

[CR35] Nardone M, Floras JS, Millar PJ (2020) Sympathetic neural modulation of arterial stiffness in humans. Am J Physiol Heart Circ Physiol. 10.1152/ajpheart.00734.2020 33035441 10.1152/ajpheart.00734.2020

[CR36] Novak P (2011) Assessment of sympathetic index from the Valsalva maneuver. Neurology 76(23):2010–2016 21646629 10.1212/WNL.0b013e31821e5563 PMC3269073

[CR37] Panerai RB, Brassard P, Burma JS, Castro P, Claassen JA, van Lieshout JJ, Liu J, Lucas SJ, Minhas JS, Mitsis GD, Nogueira RC, Ogoh S, Payne SJ, Rickards CA, Robertson AD, Rodrigues GD, Smirl JD, Simpson DM (2023) Transfer function analysis of dynamic cerebral autoregulation: a CARNet white paper 2022 update. J Cereb Blood Flow Metab 43(1):3–25. 10.1177/0271678x221119760 35962478 10.1177/0271678X221119760 PMC9875346

[CR38] Pase MP, Beiser A, Himali JJ, Tsao C, Satizabal CL, Vasan RS, Seshadri S, Mitchell GF (2016) Aortic stiffness and the risk of incident mild cognitive impairment and dementia. Stroke 47(9):2256–2261 27491735 10.1161/STROKEAHA.116.013508 PMC4995162

[CR39] Perissiou M, Bailey TG, Windsor M, Nam MCY, Greaves K, Leicht AS, Golledge J, Askew CD (2018) Effects of exercise intensity and cardiorespiratory fitness on the acute response of arterial stiffness to exercise in older adults. Eur J Appl Physiol 118:1673–1688 29850932 10.1007/s00421-018-3900-5

[CR40] Perry BG, Lucas SJ (2021) The acute cardiorespiratory and cerebrovascular response to resistance exercise. Sports Med Open 7(1):1–19 34046740 10.1186/s40798-021-00314-w PMC8160070

[CR41] Perry BG, Cotter JD, Mejuto G, Mündel T, Lucas SJ (2014a) Cerebral hemodynamics during graded Valsalva maneuvers. Front Physiol 5:1–7 25309449 10.3389/fphys.2014.00349 PMC4163977

[CR42] Perry BG, Mündel T, Cochrane DJ, Cotter JD, Lucas SJ (2014b) The cerebrovascular response to graded Valsalva maneuvers while standing. Physiol Rep 2(2):e00233 24744902 10.1002/phy2.233 PMC3966248

[CR43] Perry BG, Schlader ZJ, Barnes MJ, Cochrane DJ, Lucas SJ, Mündel T (2014c) Hemodynamic response to upright resistance exercise: effect of load and repetition. Med Sci Sports Exerc 46:479–487 23917471 10.1249/MSS.0b013e3182a7980f

[CR64] Perry BG, Bear TL, Lucas SJ, Mündel T (2016) Mild dehydration modifies the cerebrovascular response tothe cold pressor test. Exp Physiol 101(1):135–142. http://onlinelibrary.wiley.com/store/10.1113/EP085449/asset/eph1718.pdf?v=1&t=jc9wzq7f&s=bb91911ec19ec97cae7aa60339177aa05fadc391 10.1113/EP085449 26374269

[CR44] Perry BG, De Hamel T, Thomas KN, Wilson LC, Gibbons TD, Cotter JD (2020) Cerebrovascular haemodynamics during isometric resistance exercise with and without the Valsalva manoeuvre. Eur J Appl Physiol. 10.1007/s00421-019-04291-7 31912226 10.1007/s00421-019-04291-7

[CR45] Pierce DR, Doma K, Leicht AS (2018) Acute effects of exercise mode on arterial stiffness and wave reflection in healthy young adults: a systematic review and meta-analysis. Front Physiol 9:73 29487535 10.3389/fphys.2018.00073 PMC5816907

[CR46] Pott F, Knudsen L, Nowak M, Nielsen H, Hanel B, Secher N (1997) Middle cerebral artery blood velocity during rowing. Acta Physiol Scand 160(3):251–255 9246388 10.1046/j.1365-201X.1997.00144.x

[CR47] Pott F, van Lieshout JJ, Ide K, Madsen P, Secher NH (2000) Middle cerebral artery blood velocity during a Valsalva maneuver in the standing position. J Appl Physiol 88(5):1545–1550 10797110 10.1152/jappl.2000.88.5.1545

[CR48] Pott F, van Lieshout JJ, Ide K, Madsen P, Secher NH (2003) Middle cerebral artery blood velocity during intense static exercise is dominated by a Valsalva maneuver. J Appl Physiol 94(4):1335–1344 12626468 10.1152/japplphysiol.00457.2002

[CR49] Rakobowchuk M, McGowan C, De Groot P, Bruinsma D, Hartman J, Phillips S, MacDonald M (2005) Effect of whole body resistance training on arterial compliance in young men. Exp Physiol 90(4):645–651 15849230 10.1113/expphysiol.2004.029504

[CR50] Romero SA, Cooke WH (2007) Hyperventilation before resistance exercise: cerebral hemodynamics and orthostasis. Med Sci Sports Exerc 39(8):1302–1307 17762363 10.1249/mss.0b013e3180653636

[CR51] Sanchis-Gomar F, Lavie CJ, Marín J, Perez-Quilis C, Eijsvogels TM, O’keefe JH, Perez MV, Blair SN (2022) Exercise effects on cardiovascular disease: from basic aspects to clinical evidence. Cardiovasc Res 118(10):2253–2266 34478520 10.1093/cvr/cvab272

[CR52] Saz-Lara A, Cavero-Redondo I, Álvarez-Bueno C, Notario-Pacheco B, Ruiz-Grao MC, Martínez-Vizcaíno V (2021) The acute effect of exercise on arterial stiffness in healthy subjects: a meta-analysis. J Clin Med 10(2):291 33466830 10.3390/jcm10020291 PMC7831005

[CR53] Smith MM, Tony Buffington C, Hamlin RL, Devor ST (2015) Relationship between muscle sympathetic nerve activity and aortic wave reflection characteristics in aerobic-and resistance-trained subjects. Eur J Appl Physiol 115(12):2609–2619 26245524 10.1007/s00421-015-3230-9

[CR54] Swierblewska E, Hering D, Kara T, Kunicka K, Kruszewski P, Bieniaszewski L, Boutouyrie P, Somers VK, Narkiewicz K (2010) An independent relationship between muscle sympathetic nerve activity and pulse wave velocity in normal humans. J Hypertens 28(5):979–984 20408258 10.1097/hjh.0b013e328336ed9a

[CR55] Thomas HJ, Marsh CE, Naylor LH, Ainslie PN, Smith KJ, Carter HH, Green DJ (2021) Resistance, but not endurance exercise training, induces changes in cerebrovascular function in healthy young subjects. Am J Physiol Heart Circ Physiol 321(5):H881–H892 34559581 10.1152/ajpheart.00230.2021

[CR56] Tiecks FP, Lam AM, Matta BF, Strebel S, Douville C, Newell DW (1995) Effects of the valsalva maneuver on cerebral circulation in healthy adults: a transcranial Doppler study. Stroke 26(8):1386–1392 7631342 10.1161/01.str.26.8.1386

[CR57] Tomoto T, Sugawara J, Nogami Y, Aonuma K, Maeda S (2015) The influence of central arterial compliance on cerebrovascular hemodynamics: insights from endurance training intervention. J Appl Physiol 119(5):445–451 26139214 10.1152/japplphysiol.00129.2015

[CR58] Verbree J, Bronzwaer A, van Buchem MA, Daemen MJ, van Lieshout JJ, van Osch MJ (2017) Middle cerebral artery diameter changes during rhythmic handgrip exercise in humans. J Cereb Blood Flow Metab 37(8):2921–2927 27837189 10.1177/0271678X16679419 PMC5536799

[CR59] Weber T, Auer J, O’Rourke MF, Kvas E, Lassnig E, Berent R, Eber B (2004) Arterial stiffness, wave reflections, and the risk of coronary artery disease. Circulation 109(2):184–189 14662706 10.1161/01.CIR.0000105767.94169.E3

[CR60] Willie CK, Colino FL, Bailey DM, Tzeng YC, Binsted G, Jones LW, Haykowsky MJ, Bellapart J, Ogoh S, Smith KJ, Smirl JD, Day TA, Lucas SJ, Eller LK, Ainslie PN (2011) Utility of transcranial Doppler ultrasound for the integrative assessment of cerebrovascular function. J Neurosci Methods 196(2):221–237. 10.1016/j.jneumeth.2011.01.011 21276818 10.1016/j.jneumeth.2011.01.011

[CR61] Xuereb RA, Magri CJ, Xuereb RG (2023) Arterial stiffness and its impact on cardiovascular health. Curr Cardiol Rep 25(10):1337–1349 37676581 10.1007/s11886-023-01951-1

[CR62] Zhang R, Crandall CG, Levine BD (2004) Cerebral hemodynamics during the valsalva maneuver insights from ganglionic blockade. Stroke 35(4):843–847 14976327 10.1161/01.STR.0000120309.84666.AE

[CR63] Zhang Y, Zhang Y-J, Ye W, Korivi M (2021) Low-to-moderate-intensity resistance exercise effectively improves arterial stiffness in adults: evidence from systematic review, meta-analysis, and meta-regression analysis. Front Cardiovasc Med 8:738489 34708090 10.3389/fcvm.2021.738489 PMC8544752

