# Correction: Resistance exercise with combined Valsalva manoeuvre acutely increases carotid-femoral pulse wave velocity in healthy untrained individuals

**DOI:** 10.1007/s00421-026-06371-x

**Published:** 2026-07-25

**Authors:** Blake G. Perry, Melissa J. Perry, Riley Dixon, Michael Bush, Helen Ryan-Stewart

**Affiliations:** 1 School of Health Sciences, Massey University, Wallace Street, Wellington, 6021 New Zealand; 2 Medical Research Institute of New Zealand, Wellington, New Zealand; 3 Faculty of Education, Humanities and Health Sciences, Eastern Institute of Technology, Napier, New Zealand

**Correction to: European Journal of Applied Physiology**

**https://doi.org/10.1007/s00421-026-06167-z**

In the original version of this article, in the Introduction section, eighth sentence of the second paragraph, which previously read as

Heffernan et al. (2007b) reported that repeated VMs in isolation (60 total, in sets of 4) acutely reduced central PWV, but pulsatile fluctuations in MAP of similar magnitude induced by RE alone (i.e. without the VM) did not.

but should have read as

Heffernan et al. (2007b) reported that repeated VMs in isolation (60 total, in sets of 4) acutely increased central PWV, but pulsatile fluctuations in MAP of similar magnitude induced by RE alone (i.e. without the VM) did not.

The original article has been corrected.

